# Hexanoic, Octanoic and Decanoic Acids Promote Basal and Insulin-Induced Phosphorylation of the Akt-mTOR Axis and a Balanced Lipid Metabolism in the HepG2 Hepatoma Cell Line

**DOI:** 10.3390/molecules23092315

**Published:** 2018-09-11

**Authors:** Sabri Ahmed Rial, Gaetan Ravaut, Tommy B. Malaret, Karl-F. Bergeron, Catherine Mounier

**Affiliations:** Molecular Metabolism of Lipids Laboratory, BioMed Research Center, Biological Sciences Department, University of Quebec in Montreal (UQAM), Montreal, QC H3C 3P8, Canada; rial_sabri@hotmail.com (S.A.R.); gaetanravaut89@gmail.com (G.R.); tommy.malaret@gmail.com (T.B.M.); bergeron.karl-frederik@uqam.ca (K.-F.B.)

**Keywords:** medium chain fatty acids (MCFA), long chain fatty acids (LCFA), lipid metabolism, hepatocytes, lipotoxicity, insulin resistance

## Abstract

Metabolic illnesses such as non-alcoholic fatty liver disease (NAFLD) are in constant increase worldwide. Highly consumed long chain fatty acids (LCFA) are among the most obesogenic and steatogenic nutrients. Hepatic steatosis is associated with several complications such as insulin resistance. Growing evidence points to medium chain fatty acids (MCFA), more efficiently oxidized than LCFA, as a promising dietary alternative against NAFLD. However, reports on the hepatic effects of MCFA are sometimes conflicting. In this study we exposed HepG2 cells, a human hepatocellular model, to 0.25 mM of hexanoic (C6), or octanoic (C8), and decanoic (C10) acids separately or in a C8 + C10 equimolar mix reflecting commercially available MCFA-rich oils. We found that C6, a poorly studied MCFA, as well as C8 and C10 did not provoke the deleterious lipid anabolism runaway typically induced by LCFA palmitate. MCFA tended, instead, to promote a balanced metabolic profile and were generally non-cytotoxic. Accordingly, mitochondrial integrity was mostly preserved following MCFA treatment. However, treatments with C8 induced a mitochondrial membrane potential decrease, suggesting prolonged exposure to this lipid could be problematic. Finally, MCFA treatments maintained optimal insulin sensitivity and even fostered basal and insulin-dependent phosphorylation of the Akt-mTOR pathway. Overall, MCFA could constitute an effective nutritional tool to manage liver steatosis and hepatic insulin resistance.

## 1. Introduction 

Non-alcoholic fatty liver disease (NAFLD) is the most widespread chronic liver illness with a prevalence reaching 20–30% within the general population and 80–90% among obese patients [1,2]. In addition to consequences directly related to liver dysfunction, NAFLD is closely associated with several pathologies including metabolic syndrome, obesity, cardiovascular disease, insulin resistance, and type 2 diabetes [1,3]. Hepatic steatosis, the excessive accumulation of triglycerides (TG) within the liver, is the major hallmark of NAFLD. Liver steatosis is characterized by the intracellular accumulation of lipid droplets (LD) in more than 5% of cytoplasmic space [3,4,5]. At the molecular level, the pathogenesis of NAFLD is largely due to lipotoxicity [6,7]. Triglyceride accumulation in the core of LD is correlated to the intracellular accumulation of non-esterified free fatty acids and of several lipid species that are potent inducers of lipotoxicity in the liver [6,7,8,9].

Long chain fatty acids (LCFA) can trigger variable degrees of lipotoxicity depending on their saturation levels [10,11]. Saturated LCFA are the most lipotoxic. Palmitate is a 16-carbon long saturated LCFA well documented to induce markers of de novo lipogenesis, excessive LD accumulation, massive lipotoxicity, and insulin resistance in hepatocytes [12,13,14,15,16]. LCFA impede lipid oxidation and enhance reactive oxygen species (ROS) accumulation, promoting mitochondrial injury, oxidative stress, inflammation, apoptosis and necrosis [17]. Mediators of oxidative stress and inflammation exacerbate ROS accumulation and mitochondrial damages [14,17,18], and directly inhibit the activity of the insulin receptor and its substrate [19,20,21]. Lipogenesis, combined with diminished lipid catabolic activity, contribute to a steatogenic metabolic imbalance in LCFA-treated cells. 

Medium chain fatty acids (MCFA) are saturated fatty acids with chain lengths of six to ten carbons [22,23,24]. Hexanoic (C6), octanoic (C8), and decanoic (C10) acids are naturally found in coconut, palm kernel, and babassu oils where they account for around 8–18% of total fatty acids [22,23,24]. Concentrated formulas, like commercially available medium chain TG (MCT) oils, consist essentially of equimolar amounts of esterified C8 and C10, with very small amounts of C6 [24]. Because of their relatively short carbon chain length, once assimilated by organisms, MCFA undergo a distinctive metabolism compared to LCFA [25]. Dietary MCFA are transported to the liver via the hepatic portal blood system independently of chylomicron trafficking, and their translocation across mitochondrial membranes is not rate-limited by the carnitine palmitoyltransferase (CPT) system [25,26]. These properties suggest that MCFA are mainly metabolized by the liver, where they preferentially undergo mitochondrial β-oxidation instead of re-esterification [25,26]. This is supported by human trials showing that MCT intake increases postprandial oxygen consumption, energy expenditure, and fat oxidation, while improving dyslipidemia and, in some cases, adiposity [27,28,29,30,31,32,33,34]. Several molecular and animal physiology studies also support these observations. In a previous study performed on chick embryos and HepG2 hepatocytes, our team found that both C6 and C8 decreased insulin and triiodothyronine-induced fatty acid synthase (*FASN*) expression and activity [35], a key enzyme in de novo lipogenesis [36]. In L02 hepatocytes, C8 and C10 triggered less TG accumulation than LCFA. This was associated with a downregulation of the expression of key lipogenic players and an upregulation of lipolysis markers [37]. In addition, C8 and C10 did not induce the substantial inflammation caused by LCFA [38]. Similarly, high fat diets (HFD) rich in MCT did not induce obesity, insulin resistance or inflammation in mice, contrary to lard-based HFD [39]. Moreover, MCT reduced steatosis and necrosis in a rat NAFLD model [40]. 

However, less promising and sometimes conflicting results have also been reported. Mice fed an MCT-rich diet, despite exhibiting less hepatic and muscular steatosis than long chain TG-fed mice, displayed increased insulin resistance as revealed by a lowered glucose infusion rate during hyperinsulinemic euglycemic clamp [41]. The underlying mechanisms for this surprising effect are poorly understood but probably exclude pancreatic toxicity since MCFA improved β-cell functions both in vitro and in vivo [42]. In another mouse study, long-term feeding with high MCT doses led to liver steatosis, marked lipogenesis, and downregulation of β-oxidation markers, as well as mild improvement of both body mass and insulin sensitivity [43]. Hence, more studies are needed to better understand the impact of MCFA on intracellular lipid accumulation and cellular responses to insulin.

In this study, we mimicked hepatic exposure to various fatty acids by treating HepG2 cells with MCFA. C8 and C10 were administered separately or in an equimolar mix reflecting the composition of commercially available MCT oils. We also tested C6, an understudied MCFA with potent antilipogenic properties [39]. Though C8 had some impact on mitochondrial integrity, MCFA treatment did not trigger intracellular lipid accumulation or cytotoxicity. MCFA seemed instead to sustain a relative metabolic balance favoring lipid catabolism. Finally, MCFA did not impair insulin sensitivity of hepatocytes and even significantly enhanced the basal phosphorylation state of protein kinase B/Akt.

## 2. Materials and Methods

### 2.1. Cells, Chemicals and Reagents

HepG2 cells were purchased from the American Type Culture Collection (ATCC, #HB-8065). Dulbecco’s phosphate-buffered saline 1X (DPBS, #14190250), fetal bovine serum (FBS, #16000044), penicillin/streptomycin (#15140122) (10,000 U/mL and 10,000 µg/mL respectively), 3-(4,5-dimethylthiazol-2-yl)-2,5-diphenyltetrazolium bromide (MTT) Vybrant assay kit (#V13154), JC-1 dye (5,5′,6,6′-tetrachloro-1,1′,3,3′-tetraethyl-imidacarbocyanine-iodide,5,5′,6,6′-tetrachloro-1,1′,3,3′-tetraethylbenzimidazolocarbocyanine iodide) (#T3168), DAPI histone-stain dye (4′,6-diamidino-2-phenylindole dihydrochloride) (#D1306), BODIPY 493/503 lysochrome (4,4-difluoro-1,3,5,7,8-pentamethyl-4-bora-3a,4a-diaza-s-indacene) (#D3922), Trizol reagent (#15596026), and SuperScriptII RNA Reverse Transcriptase kit (#18064014), were purchased from ThermoFischer Scientific. The SensiFAST SYBR real-time PCR kit was purchased from Bioline (#BIO-98005). Poly-l-lysine solution (#P4707), oleic acid (oleate, #O1008), palmitic acid sodium salt (palmitate, # P9767), hexanoic acid sodium salt (C6; #C4026), octanoic acid sodium salt (C8; #C5038), decanoic acid sodium salt (C10; #C4151), and fatty acid-free bovine serum albumin (BSA; #A8806-1G) were purchased from Sigma-Aldrich (Saint Louis, MO, USA). Recombinant human insulin (#511-016-CM), Dulbecco’s modified Eagle medium (DMEM; 25 mM d-glucose) (#319-005-CL), and Eagle’s minimum essential medium (EMEM; 5.5 mM d-glucose) supplemented with Earle’s salts and l-glutamine (#320-005-CL), were purchased from Wisent Bioproducts (Saint-Jean-Baptiste, QC, Canada). Radiolabeled [1-14C]-oleate (#NEC317050UC) and 2-[1,2-3H(N)]-deoxy-d-glucose (#NET549250UC) were purchased from PerkinElmer (Waltham, MA, USA). 

### 2.2. Fatty Acid Conjugation to Bovine Serum Albumin 

Stocks of bovine serum albumin (BSA)-conjugated fatty acids at a 6:1 molar ratio (10 mM fatty acid: 1.7 mM BSA) were prepared as follows. Powdered palmitate (0.7%, *w*:*v*) was dissolved in 150 mM NaCl at 70 °C until the solution became clear (~1 h). Solutions of C6, C8, and C10 at 10 mM were obtained following the same procedure, except that their dissolution in 150 mM NaCl was achieved at 37 °C. Fatty-acid free BSA (0.12%, *w*:*v*) was dissolved in 150 mM NaCl at 37 °C. These fatty acids and BSA solutions were then slowly mixed at a 1:1 volume ratio and stirred 1 h at 37 °C, pH was adjusted to 7.4, and the final stock solutions were sterile-filtered. 

### 2.3. Cell Culture 

Unless otherwise stated, HepG2 human hepatocellular carcinoma cells were seeded in regular non-pyrogenic plates at 5.2 × 10^4^ cells/cm^2^ and cultivated in EMEM supplemented with FBS (10%), penicillin (100 U/mL) and streptomycin (100 µg/mL) under standard conditions (37 °C, 5% CO_2_) until cells reached ~80% confluence. Cells were then transferred FBS-free EMEM and starved 24 h before a 24 h treatment with either palmitate, C6, C8, C10 or an equimolar mix of C8 + C10. 

### 2.4. MTT Cell Viability Assay 

Cell viability was assessed by the reduction of yellow water-soluble tetrazolium salt into the blue non-water-soluble formazan [44,45]. Treated cells were exposed to FBS-free EMEM supplemented with 1.2 mM MTT reagent for 3 h at 37 °C. A lysis solution (10% SDS, 0.01 mM HCl) was mixed into the medium and plates were incubated 4 h at 37 °C. The absorbance of cell lysates (570 nm) was measured using the BioTek Gen5 software and the Eon Biotek plate reader. Viability percentage was calculated by reporting the absorbance of a fatty acid treated sample with the absorbance of a corresponding BSA-treated sample. 

### 2.5. Mitochondrial Membrane Potential Measurement 

The JC-1 dye was used to evaluate the mitochondrial integrity of HepG2 cells. Fluorescence emission at 585 nm is indicative of JC-1 aggregation and optimal mitochondrial membrane potential [46,47]. Fatty acid-treated cells were resuspended in prewarmed EMEM at 2.5 × 10^5^ cells/mL supplemented with JC-1 dye (1 µg/mL). After 30 min of incubation at 37 °C in the dark, cells were centrifuged (3 min, 150 g) and resuspended again in EMEM, then processed through a BD Facs Jazz cell sorter using an excitation laser at 488 nm. Fluorescence intensity at 585 nm (5000 events) was considered proportional to mitochondrial membrane potential integrity. 

### 2.6. Lipid Droplet Staining 

HepG2 cells grown on poly-l-lysine coated slides were washed with ice-cold DPBS and fixed in 3% paraformaldehyde for 30 min at room temperature. Cells were then treated 10 min with a 120 mM NaCl solution containing both DAPI (1 μg/mL) and BODIPY 493/503 (1 μg/mL) for nuclei and LD staining, respectively. Fluorescence was visualized with a Nikon A1 confocal microscope, and image stacks covering the entire cell thickness (z-axis) were taken using the NIS Element software. Finally, image stack projections were analyzed with FIJI software to count nuclei and to measure LD number and size (µm^2^). 

### 2.7. Western Blot Analysis

Cells were washed with ice-cold DPBS and total proteins were extracted using radioimmunoprecipitation assay buffer (50 mM Hepes, 125 mM NaCl, 100 mM NaF, 10 mM Na-pyrophosphate, 10% glycerol, 1% Triton X-100, 1.5 mM MgCl_2_, 1 mM ethylene glycol-bis(2-aminoethylether)-*N*,*N*,*N*′,*N*′-tetraacetic acid, 2 mM Na-orthovanadate, 1.5 mM PMSF, 1× cOmpete protease inhibitors, pH 7.2). Protein concentration was determined by the Bradford method [48], then diluted at 1 µg/µL in Laemmli buffer (50 mM Tris-HCl at pH 6.8, 5% β-mercaptoethanol, 2% sodium dodecyl sulfate (SDS), 0.01% bromophenol blue, 10% glycerol) before denaturation by heating of 5 min at 95 °C. Ten µg of denatured proteins were loaded onto SDS-polyacrylamide gel electrophoresis, and immunoblot analyses were carried out using the primary antibodies listed in Table 1. Horseradish peroxidase (HRP)-conjugated anti-rabbit IgG (Abcam, #ab6721) was used as secondary antibody at 1:5000. Bands were visualized with a chemiluminescent HRP substrate (Millipore, WBKLS0500). Bands intensities were measured by densitometric analysis using FIJI software.

### 2.8. Quantitative Reverse Transcriptase-Polymerase Chain Reaction (RT-PCR) Analysis

Total cellular RNA was extracted using the Trizol reagent, following the manufacturer’s instructions. Subsequently, 1 µg of total RNA was reverse-transcribed into cDNA using the SuperScript II kit before quantitative PCR analysis using the SensiFAST SYBR real-time PCR kit and the LightCycler 480 Real-Time PCR System (Roche Applied Science, Laval, QC, Canada). Metabolic genes of interest and the *HPRT1* house-keeping gene were amplified with the primers listed in Table 2. To avoid amplification of genomic DNA, primer pairs were systematically designed to span at least one intron. Gene expression was calculated based on the comparative ∆Ct method.

### 2.9. Oleate Oxidation Assay 

The oxidation rate of radiolabeled [^14^C]-oleate into ^14^CO_2_ was measured as previously described [49,50], with minor modifications. Briefly, cells were seeded in 6-well plates at 5.2 × 10^4^ cells/cm^2^, then treated 24 h with fatty acids once reaching 80% of confluency. Afterward, cells were exposed 1 h to preincubation buffer (DMEM containing 12 mM d-glucose, 4 mM glutamine, 25 mM Hepes, 1% fatty acid-free BSA, 0.25 mM oleate). The preincubation buffer was then supplemented with (^14^C)-oleate (1 µCi/mL and a small disk of Whatman paper #3 (3.5 mm) wetted with 3 M NaOH was affixed to the top of the well. After a 1.5 h incubation (37 °C, 5% CO_2_), 0.5 mL of 70% perchloric acid was added into the cell medium. Gaseous ^14^CO_2_ released from the cell medium over 1 h (room temperature) was trapped in the Whatman paper and used for liquid scintillation counting using a Tri-Carb 2800T liquid scintillation analyzer with QuantaSmart software. Parallel plates undergoing the same treatments were used to measure protein concentration and normalize counts per minutes (CPM/µg protein). 

### 2.10. Glucose Uptake Assay 

Cellular uptake of radiolabeled [^3^H]-deoxy-d-glucose in response to insulin was measured as described previously [51,52], with minor modifications. Cells treated with fatty acids were stimulated 1 h with 100 nM insulin. Then, cells were washed twice with pre-warmed (37 °C) Krebs-Ringer buffer (118 mM NaCl, 4.8 mM KCl, 1.2 mM KH_2_PO_4_, 1.2 mM MgSO_4_, 2.5 mM NaHCO_3_, 11 mM d-glucose, 0.07% fatty acid-free BSA, pH 7.4) prior to 20 min incubation in Krebs-Ringer buffer supplemented with (^3^H)-deoxy-d-glucose (0.5 µCi/mL). Plates were then placed on ice and the cells were washed three times with ice-cold DPBS. Cells were finally homogenized in lysis buffer (1% NP-40, 0.1% SDS) and the homogenate was used for both Bradford protein dosage and liquid scintillation counting using a Tri-Carb 2800T liquid scintillation analyzer with QuantaSmart software. Disintegrations per minutes (DPM) were then normalized to protein concentration (DPM/µg protein).

### 2.11. Statistical Analysis

Data are presented as mean ± standard error of the mean (SEM). A Student’s *t*-test was used to evaluate statistical significance. Generally, a one-tailed unpaired test was applied, except with data normalized to control where a “one sample” *t*-test is appropriate. A *p*-value < 0.05 was considered statistically significant. Each result has been obtained from at least three distinct cell cultures prepared on different days (*n* ≥ 3 independent replicates).

## 3. Results

### 3.1. Medium Chain Fatty Acids Are Not Cytotoxic but Induce Differential Effects on Mitochondria 

To determine the effects of MCFA on hepatocyte viability, we exposed HepG2 cells to increasing doses (0.1–0.5 mM) of either palmitate or various MCFA before an MTT reduction assay. For reference, circulating fatty acids range between 0.2 mM and 2 mM in physiological conditions [9,53,54]. 

**Figure 1:**
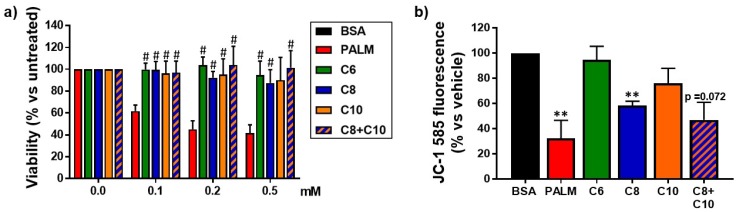
Effect of fatty acids on HepG2 cell viability and mitochondrial membrane potential. HepG2 cells were treated for 24 h with palmitate (PALM), C6, C8, C10, a C8 + C10 equimolar mix, or vehicle control (BSA). (**a**) Quantitative analysis of MTT cell viability assay results (*n* = 3) relative to untreated cells (0.0 mM). # *p*-value < 0.05 versus palmitate treatment. (**b**) Mitochondrial membrane potential relative to vehicle-treated control, as measured with the JC-1 dye (*n* = 3). ** *p*-value < 0.01 versus BSA control.

While palmitate decreased cell viability in a dose-dependent manner, equivalent doses of various MCFA did not alter it (Figure 1a). Since the reduction of the MTT reagent is dependent on mitochondrial electronic flux [45], Figure 1a also indicates that, contrary to palmitate, MCFA treatments maintained this mitochondrial function.

Since mitochondrial membrane permeability and respiratory chain can both be damaged in cells exposed to free fatty acids [17,55,56], we compared the impact of a 24 h exposure to 0.25 mM MCFA or palmitate on mitochondrial membrane integrity using JC-1 fluorescence. As expected, palmitate strongly decreased average mitochondrial membrane potential. Incubation of cells with either C6 or C10 did not significantly damage mitochondrial membrane potential. However, C8 alone or in combination with C10 unexpectedly decreased it to an extent nearing the effect of palmitate (Figure 1b).

### 3.2. Medium Chain Fatty Acids Do Not Trigger Intracellular Lipid Storage or De Novo Lipogenesis 

**Figure 2:**
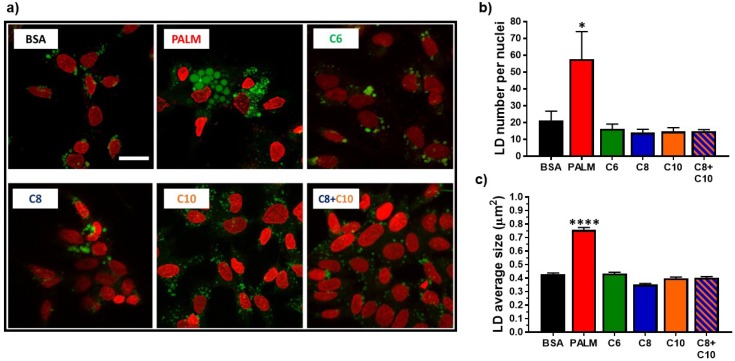
Effect of fatty acids on HepG2 intracellular lipid storage. HepG2 cells were treated 24 h with 0.25 mM PALM, C6, C8, C10, a C8 + C10 equimolar mix, or vehicle control (BSA). (**a**) Representative images of LD staining (green). Cell nuclei are shown in red. Scale bar = 20 µm. (**b**) LD number per cell nuclei (*n* = 4). (**c**) Average LD size in µm^2^ (*n* = 4). * *p*-value < 0.05, **** *p*-value < 0.0001 versus BSA control.

We next compared the effects of MCFA and LCFA on intracellular lipid storage. Unsurprisingly, palmitate potently increased the number (3-fold) and size (2-fold) of LD (Figure 2a–c). Moreover, palmitate substantially expanded the range of LD size (Figure S1a). In contrast, treating cells with MCFA did not induce modifications of LD characteristics (Figure 2 and Figure S1a). 

**Figure 3:**
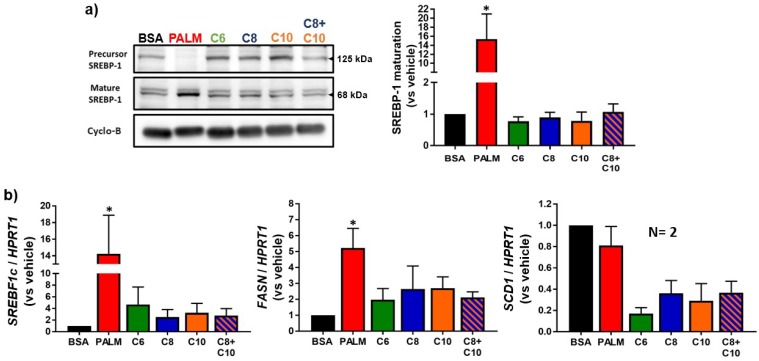
Effect of fatty acids on HepG2 lipid anabolism. HepG2 cells were treated 24 h with 0.25 mM PALM, C6, C8, C10, a C8 + C10 equimolar mix, or vehicle control (BSA). (**a**) Representative Western blots of precursor and mature (cleaved) SREBP-1 isoforms, as well as Cyclophilin-B loading control, and quantification of SREBP-1 maturation (mature/total ratio) relative to vehicle-treated control (*n* = 3). (**b**) *SREBF1c*, *FASN* and *SCD1* lipogenic marker mRNA expression relative to vehicle-treated control (*n* = 3). * *p*-value < 0.05 versus BSA control.

Palmitate is known to increase lipid storage by triggering lipid synthesis in hepatocytes [10,57], a process mainly controlled by the SREBP-1 transcription factor [36]. Not surprisingly, our analyses revealed that incubation with palmitate induced SREBP-1 maturation (Figure 3a). Accordingly, the expression of lipogenic genes (and SREBP-1 targets) *SREBF1c* and *FASN* was strongly induced by palmitate treatment (Figure 3b). Despite the transcriptional overexpression of *SREBF1c* (Figure 3b), abundance of the SREBP-1 precursor encoded by this gene was lowered (Figure 3a), suggesting massive proteolytic activation under palmitate treatment. In contrast, none of the MCFA tested had a significant effect on SREBP-1 maturation (Figure 3a). Interestingly, the expression of *SCD1*, though not substantially modulated by palmitate, was drastically downregulated by MCFA treatments (Figure 3b). Though it was not statistically significant, treatments including C10 tended to increase transcriptional expression of *PPARG* (Figure S1b), a gene mainly involved in fatty acid uptake and storage [58]. 

### 3.3. Medium Chain Fatty Acids Sustain Lipid Catabolism 

**Figure 4:**
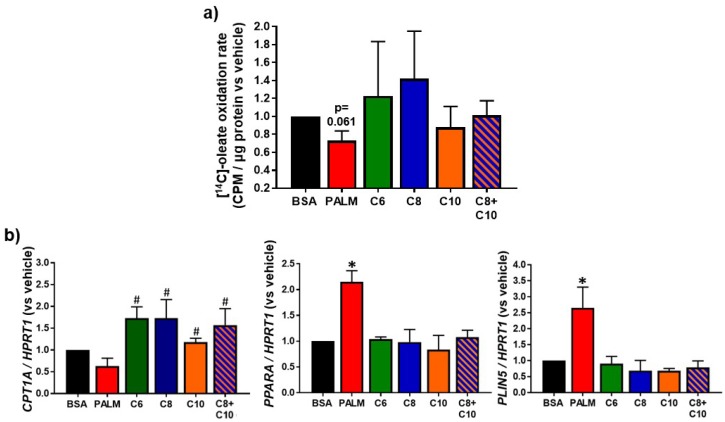
Effect of fatty acids on HepG2 lipid β-oxidation activity. HepG2 cells were treated 24 h with 0.25 mM PALM, C6, C8, C10, a C8 + C10 equimolar mix, or vehicle control (BSA). (**a**) (^14^C)-oleate oxidation assay results relative to vehicle-treated control (*n* = 4). (**b**) *CPT1A, PPARA,* and *PLIN5* lipid catabolism marker mRNA expression relative to vehicle-treated control (*n* = 3). * *p*-value < 0.05 versus BSA control, # *p*-value < 0.05 versus palmitate treatment.

To complete our analysis of the lipid metabolism profile, we compared the effects of MCFA and LCFA on lipid catabolism. To do so, we measured the oleate oxidation rate. We also determined the expression levels of *CPT1A*, *PPARA,* and *PLIN5*, genes involved in the mitochondrial β-oxidation process [59,60,61]. While palmitate tended to cause a decrease in fatty acid oxidation, MCFA did not (Figure 4a). We rather observed a trend towards higher oleate oxidation with C6 and C8 (Figure 4a). Consistent with these distinct results, *CPT1A* expression followed a similar pattern: Palmitate tended to decrease the expression of *CPT1A* while all MCFA treatments tended to increase it, with weaker effect from C10 (Figure 4b). Of note, all MCFA resulted in increased *CPT1A* expression relative to palmitate. *PPARA* expression was unexpectedly increased by palmitate but remained unchanged following MCFA treatment (Figure 4b). Interestingly, expression of *PLIN5,* which encodes for a protein involved in ld-mitochondria linkage for fat utilization [61,62], followed the same pattern as *PPARA* (Figure 4b). 

### 3.4. Medium Chain Fatty Acids Maintain Insulin Sensitivity and Foster Akt-mTOR Axis Activation 

**Figure 5:**
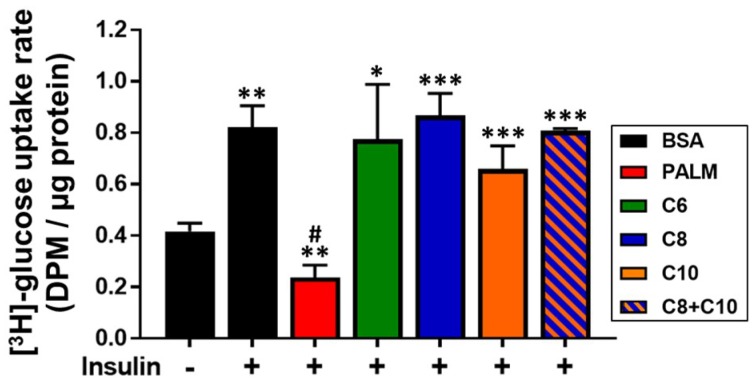
Effect of fatty acids on insulin-induced glucose uptake by HepG2 cells. HepG2 cells were treated 24 h with 0.25 mM PALM, C6, C8, C10, a C8 + C10 equimolar mix, or vehicle control (BSA). (^3^H)-deoxy-d-glucose uptake assay results (*n* = 3) were obtained after a 1 h-long stimulation with 100 nM insulin (+). * *p*-value < 0.05, ** *p*-value < 0.01, *** *p*-value < 0.001 versus non-stimulated BSA control. # *p*-value < 0.05 versus stimulated BSA control.

Lipid deposition in hepatocytes typically leads to insulin resistance [13,63]. As MCFA treatment did not lead to lipid deposition, we reasoned that it might not trigger insulin resistance either. To explore this possibility, we measured the cellular glucose uptake rate in response to insulin stimulation (100 nM, 1 h). As expected [13,64], palmitate drastically impaired insulin-induced glucose uptake, below the non-stimulated level (Figure 5). However, MCFA preserved normal cellular response to insulin (Figure 5). 

**Figure 6:**
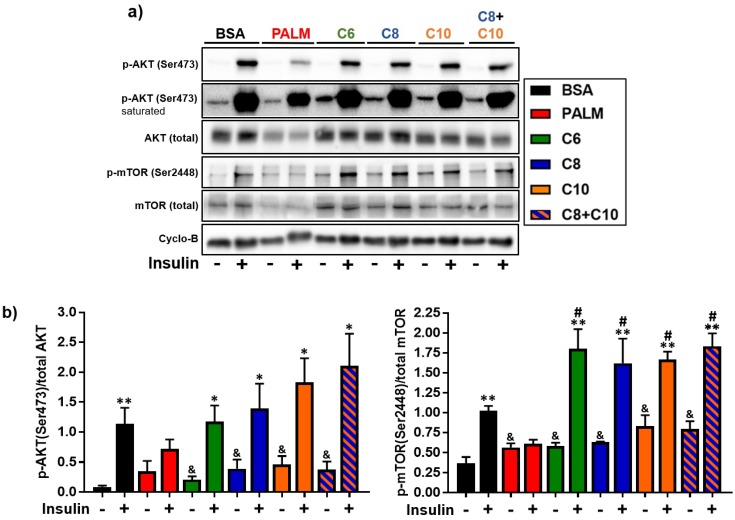
Effect of fatty acids on the insulin-induced signaling cascade in HepG2 cells. HepG2 cells were treated 24 h with 0.25 mM PALM, C6, C8, C10, a C8 + C10 equimolar mix, or vehicle control (BSA) then stimulated (+), or not (−), with 100 nM insulin for 10 min. (**a**) Representative Western blots of total and phosphorylated Akt (Ser473), total and phosphorylated mTOR (Ser2448), and Cyclophilin-B loading control (*n* = 4). (**b**) Quantification of phosphorylation levels normalized to total levels. * *p*-value < 0.05, ** *p*-value < 0.01 < 0.001 versus non-insulin stimulated. # *p*-value < 0.05 versus insulin-stimulated BSA control. & *p*-value < 0.05 versus non-insulin stimulated BSA control.

We also measured the expression and phosphorylation level of two key proteins involved in the insulin signaling pathway in fatty acid-treated hepatocytes. The phosphorylation of Akt (Ser473) and mTOR (Ser2448) was evaluated in the basal state and after insulin stimulation (100 nM, 10 min). Palmitate diminished total Akt and total mTOR expression levels (Figure 6a). This observation is consistent with the fact that palmitate is known to trigger proteasomal degradation of insulin signaling proteins [63]. Moreover, both Akt and mTOR became non-responsive to insulin stimulation after palmitate treatment (Figure 6), as expected from the literature [63,65]. On the other hand, MCFA treatments preserved the expression levels of both kinases and the normal induction of Akt phosphorylation in response to insulin. MCFA also significantly increased the insulin-dependent phosphorylation of mTOR (by more than a 1.5-fold relative to insulin-stimulated BSA control). At the same time, MCFA enhanced the basal phosphorylation of both kinases. 

## 4. Discussion

In this study, we compared the effects of three distinct MCFA with palmitate, a well-characterized LCFA known for its lipotoxic effects [17,18,19]. Our study was exclusively conducted on HepG2 cells, a cell line derived from a hepatocellular carcinoma. Therefore, care should be taken in the extrapolation of our results to hepatocytes in vivo. As expected, palmitate damaged mitochondrial function and induced dose-dependent cell mortality (Figure 1), promoted lipid accumulation (Figure 2 and Figure 3), impaired lipid catabolism (Figure 4), and triggered insulin resistance (Figure 5 and Figure 6). In a manner consistent with previous reports [35,37,40], exposure to 0.25 mM MCFA did not induce cell mortality (Figure 1a) or deleterious fat accumulation (Figure 2). Interestingly, MCFA innocuity seemed to be mediated, at least in part, by strong inhibition of *SCD1* expression (Figure 3b); not only by non-activation of SREBP-1 and subsequent lipogenesis. SCD1-synthesized monounsaturated fatty acids, mainly oleate and palmitoleate, are major substrates for TG synthesis [4,66]. Inhibition of SCD1 expression might, therefore, underlie the absence of TG accumulation in MCFA-treated cells. A similar decrease in *SCD1* expression was reported following treatment of 3T3-L1 adipocytes with C8 [67]. The effect of MCFA on *SCD1* expression should be validated in a physiological context, in liver and adipose tissues. 

### 4.1. Differential Effects of MCFA on Mitochondria

Exposure to palmitate is known to trigger oxidative stress that in turn impedes mitochondrial integrity and functions [14,68,69]. Accordingly, palmitate dramatically decreased the mitochondrial membrane potential of HepG2 cells (Figure 1b). Though MCFA are readily metabolized, they do not induce cytotoxic ROS accumulation or damages related to oxidative stress [70]. Correspondingly, C6 and C10 preserved mitochondrial membrane integrity. However, C8 administered alone or in combination with C10 unexpectedly impaired it (Figure 1b). C8 induced a mitochondrial membrane potential decrease that seemed insufficient to diminish mitochondrial activity, as MTT reduction (in the context of our cell viability assays) was not affected (Figure 1a). Indeed, a drop in mitochondrial membrane potential does not necessarily lead to the loss of mitochondrial functions [71]. As MCT have been found to promote mitochondrial biogenesis [43,72,73,74], one possibility is that an increased number of mitochondria compensate for a less optimal membrane potential. Of note, exposure to C8 has been reported to cause some apoptosis in L02 hepatocytes, though at a much lower extent than LCFA [38]. It seems C8 can potentially have deleterious impacts on mitochondrial activity and/or hepatocyte viability under certain conditions. 

### 4.2. Lipid Catabolism in MCFA-Treated Hepatocytes

MCFA-treated hepatocytes exhibited normal lipid anabolism, as shown by the measure of lipid synthesis markers and LD parameters (Figure 2 and Figure 3). Palmitate-treated hepatocytes, however, displayed enhanced lipogenic gene expression and attenuated lipid catabolism (Figure 3 and Figure 4), an imbalance that was associated with lipid accumulation (Figure 2). Consistent with their propensity towards rapid oxidation [23,25,26] and their antilipogenic effects [35], MCFA did not stimulate the cellular lipogenic process. Contrary to palmitate, MCFA did not impair the oxidation of exogenous oleate (Figure 4a). We speculate that CPT1A, the rate-limiting enzyme for mitochondrial β-oxidation [75], could be more efficient in MCFA-treated cells relative to palmitate, as malonyl-CoA (a de novo lipogenesis intermediate) is a potent inhibitor of the CPT system [76,77]. Moreover, C6- and C8-treated hepatocytes displayed higher expression of *CPT1A* (Figure 4b) than palmitate-treated cells. Increased *CPT1A* expression could result from mitochondria multiplication following MCFA exposure [43,72,73,74]. Interestingly, the effects of C10 and of the C8 + C10 mixture on oleate oxidation appeared weaker than for the other MCFA, and this was reflected in a weaker induction of *CPT1A* expression (Figure 4). In a neuronal cell model, C10 β-oxidation was recently proposed to be dependent on *CPT1A* [78]. This challenges the common assertion that all MCFA are readily oxidized, independently of the CPT system. Putative competition between oleate and C10 for mitochondrial translocation by CPT1A might underscore the tendency towards weaker oleate oxidation observed in C10-treated cells. Overall, MCFA appear to sustain a lipid metabolism balance favoring catabolism in hepatocytes, especially compared to palmitate. 

MCFA treatment did not stimulate *PPARA* gene expression while palmitate did so quite efficiently (Figure 4b). This observation was unexpected since *PPARA* is typically associated with increased lipid catabolism [79]. Dietary MCT enhance hepatic *PPARA* overexpression [80] and nuclear PPARα activity [40] in rodents, in correlation with increased lipid oxidation [40]. In fact, *CPT1A* [60] as well as *PLIN5* and *PPARA* itself [62,81,82], are known transcriptional targets of PPARα (the protein encoded by *PPARA*) [60]. Accordingly, *PLIN5* followed the same pattern of expression as *PPARA* (Figure 4b). Our contrasting in vitro data may indicate that the influence of MCFA on *PPARA*-dependent catabolic responses does not only involve hepatocytes and that a more complex mechanism is at play in physiologic systems. Our data suggest that MCFA induce *CPT1A* expression independently of PPARα in hepatocytes (Figure 4). A similar *PPARA*-independent induction of *CPT1A* expression by C8 and C10 has also been reported in L02 hepatocytes [37]. *PPARA* overexpression in palmitate-treated cells could result from de novo lipogenesis since our data suggest that palmitate increased this pathway (Figure 3). In fact, some intracellular lipogenesis intermediates activate PPARα [79]. In our study, MCFA did not activate lipogenesis nor did they stimulate *PPARA* expression. 

### 4.3. Akt-mTOR Phosphorylation in MCFA-Treated Hepatocytes

The Akt-mTOR axis is central to the insulin signaling cascade [83]. In our experiments, basal phosphorylation of Akt and mTOR was enhanced by all MCFA treatments (Figure 6). Basal Akt phosphorylation has been reported to be elevated in the livers of obese insulin-resistant mice [84]. However, elevated basal phosphorylation of Akt has also been correlated to improved insulin sensitivity in hepatocytes [63,85]. Basal Akt phosphorylation is therefore not necessarily indicative of insulin signaling activity. In our study, we observed lower insulin-induced Akt phosphorylation (Figure 6) and glucose uptake (Figure 5) after palmitate treatment. Importantly, we confirmed that Akt phosphorylation, as well as the insulin response, were preserved in MCFA-treated hepatocytes (Figure 5 and Figure 6). This result is consistent with several in vivo studies where MCT-based HFD improved fasting glycemia, insulinemia, glucose tolerance, and insulin sensitivity [39,86,87]. Though insulin-stimulated phosphorylation of mTOR was increased following MCFA treatment, insulin-stimulated phosphorylation of Akt (at Ser473) was not significantly so (Figure 6), perhaps because our robust stimulation protocol (100 nM, 10 min) saturated the phosphorylation of Akt. A hypothetical model we propose is that MCFA are readily oxidized in cells and stimulate the mTOR energy-sensing pathway [88], as reflected by the enhanced basal phosphorylation of mTOR (Figure 6b). The mTOR complex 2, directly activated by the mTOR complex 1, could then exert its positive feedback and enhance the basal phosphorylation of Akt [88,89,90]. While this proposed mechanism could explain an MCFA-triggered increase in basal Akt and mTOR phosphorylation, it does not explain the selective enhance in insulin-stimulated phosphorylation of mTOR. Further studies should be conducted in vivo to better characterize the effects of MCFA on hepatic insulin pathway under physiological conditions. 

## 5. Conclusions

This study was designed to compare the effects of MCFA with palmitate, a representative LCFA, on lipid metabolism and insulin sensitivity in a hepatocellular model. We found that C6, C8, and C10 induced no deleterious lipid accumulation and no insulin resistance. In comparison, palmitate strongly impaired both lipid homeostasis and insulin sensitivity. As dietary lipids, MCFA could constitute a healthier alternative to LCFA, potentially preventing hepatic steatosis development. Care should be taken with C8 considering its potential impact on mitochondrial function. Of great interest, MCFA could effectively represent an interesting tool to manage insulin resistance, as they appear to potentiate the Akt-mTOR pathway in hepatocytes.

## Figures and Tables

**Table 1:** Primary antibodies used for Western blots.

Antibody Target	Manufacturer	Catalog Number	Concentration Used
SREBP-1	Santa Cruz Biotechnology	#sc-8984	1:2000
p-Akt (Ser473)	Cell Signaling Technology	#4060	1:1000
p-mTOR (Ser2448)	Cell Signaling Technology	#2971	1:1000
Akt (pan)	Cell Signaling Technology	#4691	1:1500
mTOR	Cell Signaling Technology	#2972	1:1000
Cyclophilin-B	Abcam	#ab16045	1:50,000

**Table 2:** Oligonucleotides used for quantitative RT-PCR analysis.

Gene Target	Forward Primer (5′-3′)	Reverse Primer (5′-3′)
*SREBF1c*	ACAGTGACTTCCCTGGCCTAT	GCATGGACGGGTACATCTTCAA
*CPT1A*	ATCAATCGGACTCTGGAAACGG	TCAGGGAGTAGCGCATGGT
*PLIN5*	AAGGCCCTGAAGTGGGTTT	GCATGTGGTCTATCAGCTCCA
*PPARA*	CGGTGACTTATCCTGTGGTCC	CCGCAGATTCTACATTCGATGTT
*FASN*	AAGGACCTGTCTAGGTTTGATGC	TGGCTTCATAGGTGACTTCCA
*SCD1*	TTCCTACCTGCAAGTTCTACACC	CCGAGCTTTGTAAGAGCGGT
*HPRT1*	CCTGGCGTCGTGATTAGTGAT	AGACGTTCAGTCCTGTCCATAA

## Supplementary Materials

The following are available online. Figure S1: Lipid droplet size distribution and PPARG expression in MCFA-treated HepG2 cells.
