# Supplementary material for: Hexanoic, Octanoic and Decanoic Acids Promote Basal and Insulin-Induced Phosphorylation of the Akt-mTOR Axis and a Balanced Lipid Metabolism in the HepG2 Hepatoma Cell Line
Source: Molecules. 2018 Sep 11;23(9):2315. doi: 10.3390/molecules23092315 (PMC6225498; doi:10.3390/molecules23092315)
Supplement: Supplementary file 1 [file molecules-23-02315-s001.pdf]

Supplementary material

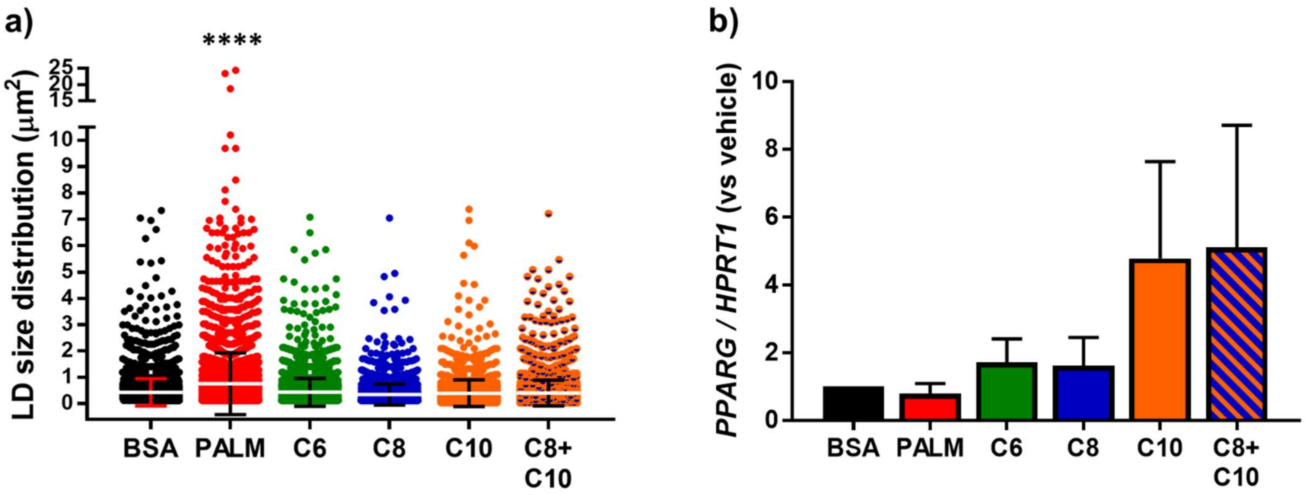

Supplementary figure 1. Lipid droplet size distribution and PPARG expression in MCFA-treated HepG2 cells.

a) Once treated with fatty acids, HepG2 cells were processed for lipid droplet (LD) staining, imaging and analysis as described in section *2.6* of the article. Data are presented as mean ± standard deviation. A one-tailed unpaired Student's t-test was used to evaluate statistical significance. A p-value < 0.05 was considered statistically significant (n = 4 independent replicates). ****, p-value ≤ 0.0001.

b) Gene expression of *PPARG* have been evaluated following the procedure described in section *2.8* of the article, using primers PPARG_Fwd (TCTCTCCGTAATGGAAGACG) and PPARG_rev (GCATTATGAGACATCCCCAC). Data are presented as mean ± standard error of the mean (SEM). A "one sample" t-test was used to evaluate statistical significance. A p-value < 0.05 was considered statistically significant (n = 3 independent replicates).
